# 手性非水毛细管电泳法测定沙美特罗替卡松粉吸入剂中昔萘酸沙美特罗对映体

**DOI:** 10.3724/SP.J.1123.2021.06002

**Published:** 2021-12-08

**Authors:** Xu ZHANG, Miaoxue DONG, Yin XU, Lijuan WANG, Xiaoqiang QIAO

**Affiliations:** 1.河北省药物质量分析控制重点实验室, 河北大学生命科学与绿色发展研究院, 河北大学药学院, 河北 保定 071002; 1. Key Laboratory of Pharmaceutical Quality Control of Hebei Province, Institute of Life Science and Green Development, College of Pharmaceutical Sciences, Hebei University, Baoding 071002, China; 2.天津阿尔塔科技有限公司, 天津 300457; 2. Tianjin Alta Scientific Co., Ltd., Tianjin 300457, China

**Keywords:** 非水毛细管电泳, L(+)-酒石酸-硼酸络合酸, 对映体, 沙美特罗替卡松粉吸入剂, nonaqueous capillary electrophoresis (NACE), L(+)-tartaric acid-boric acid complex, enantiomer, salmeterol fluticasone powder inhalant

## Abstract

昔萘酸沙美特罗是目前治疗哮喘夜间发作和哮喘维持治疗的理想药物之一,它在临床上以外消旋体形式给药。昔萘酸沙美特罗的两个对映体在药理活性和毒理作用等方面差异较大,建立昔萘酸沙美特罗对映体的手性分离分析方法对提高手性药物质量、保证临床用药安全有效具有重要意义。该文以L(+)-酒石酸-硼酸络合酸为手性选择剂,建立了测定沙美特罗替卡松粉吸入剂中昔萘酸沙美特罗对映体含量的非水毛细管电泳法。实验考察了L(+)-酒石酸浓度、硼酸浓度和表观pH(apparent pH, pH^*^ )对手性分离效果的影响。优化的缓冲溶液为:含120.0 mmol/L L(+)-酒石酸和120.0 mmol/L硼酸的甲醇溶液,pH^*^ 为0.93;其他实验条件为:未涂层弹性熔融石英毛细管(内径50.0 μm,总长度64.5 cm,有效长度55.5 cm),重力进样17.5 cm×10.0 s,检测波长225 nm,室温,工作电压20.0 kV。在优化的实验条件下,昔萘酸沙美特罗的两个对映体在18.0 min内获得了2.18的分离度;在27.5~800.0 mg/L质量浓度范围内,与峰面积呈现良好的线性关系,相关系数(*r*)大于0.9990;检出限和定量限分别为7.5和25.0 mg/L;加标回收率为98.1%~101.9%,相对标准偏差为1.2%~1.9%。随机购买市面上出售的沙美特罗替卡松粉吸入剂,对其昔萘酸沙美特罗对映体的含量进行了分析检测。结果显示,昔萘酸沙美特罗对映体1和对映体2的标示量百分含量均为98.7%, RSD分别为2.5%和2.7%。该方法操作简便易行,结果准确可靠,消耗低,可用于市售沙美特罗替卡松粉吸入剂中昔萘酸沙美特罗对映体的含量测定。

## Abstract (en)

Salmeterol xinafoate (SalX) is one of the ideal drugs used for the treatment of nocturnal asthma attacks and daily maintenance. The molecular structure of SalX contains a chiral carbon atom, and thus, SalX has two enantiomers, viz. (*R*)-SalX and (*S*)-SalX. It is clinically administered in the racemic form. Related studies have shown that the two enantiomers of SalX are quite different in pharmacology, toxicology, and other aspects. Therefore, it is of great significance to establish an analytical method for the chiral separation and determination of the SalX enantiomers to guarantee their quality and ensure their safety and effectiveness in clinical use. In this study, a chiral nonaqueous capillary electrophoresis (NACE) method, using a L(+)-tartaric acid-boric acid complex as the chiral selector, was established to determine the enantiomers of SalX in salmeterol fluticasone powder inhalant. The L(+)-tartaric acid-boric acid complex was synthesized in situ by the reaction of L(+)-tartaric acid and boric acid in methanol solution. The ion pair principle was considered the enantioseparation mechanism, and the non-aqueous system was found to be more favorable for ion pair formation, which is useful for chiral recognition. Chiral separation is based on the reversible formation of diastereomeric ion pairs between the negatively charged L(+)-tartaric acid-boric acid complex and the positively charged salmeterol enantiomers. Due to the difference in ion-pair binding ability between different enantiomers, the apparent electrophoretic mobilities of different enantiomers were also different, resulting in chiral separation in NACE. To achieve good resolution, the effects of L(+)-tartaric acid concentration, boric acid concentration, and apparent pH (pH^*^ ) on the chiral separation were investigated. The optimized buffer solution (pH^*^ 0.93) contained 120.0 mmol/L L(+)-tartaric acid and 120.0 mmol/L boric acid in methanol. Other experimental conditions were as follows: uncoated fused-silica capillary with an I. D. of 50.0 μm, a total length (*L*_tot_) of 64.5 cm, and an effective length (*L*_eff_) of 55.5 cm, along with gravity injection of 17.5 cm×10.0 s, detection wavelength of 225 nm, room temperature, and operating voltage of 20.0 kV. Under these experimental conditions, the two enantiomers of SalX achieved a resolution of 2.18 within 18.0 min. Both enantiomers showed a good linear relationship of the peak area in the concentration range of 27.5-800.0 mg/L, the correlation coefficient (*r*) being greater than 0.9990. The detection limit (*S/N*=3) and quantitative limit (*S/N*=10) were 7.5 mg/L and 25.0 mg/L, respectively; the standard recovery was 98.1%-101.9%, with relative standard deviations (RSDs) of 1.2%-1.9%. The intra- and inter-day precisions were examined, and the RSDs of the peak area and migration time were found to be below 4.9% and 1.9%, respectively, indicating good repeatability (inter-day) and reproducibility (inter-day) of the method. The established chiral NACE method was used to determine the two SalX enantiomers in a random salmeterol fluticasone powder inhalant purchased from a local market. The results showed that the percentage of labeled quantities was 98.7% for both enantiomer 1 and enantiomer 2, with RSDs of 2.5% and 2.7%, respectively. Thus, this method is simple, feasible, accurate, and inexpensive, and can be applied for the determination of SalX enantiomers in commercially available salmeterol fluticasone powder inhalants.

昔萘酸沙美特罗(salmeterol xinafoate, SalX)是一种药效持久的*β*_2_-肾上腺受体兴奋剂,药理活性好,是目前临床上治疗哮喘病的理想药物之一^[1]^。如图1所示,昔萘酸沙美特罗的分子结构中含有一个手性碳原子,有(*R*)-SalX和(*S*)-SalX两个对映体。相关研究^[2]^发现,它们的药理活性差异很大,后者对*β*_2_-肾上腺受体的选择性比前者高4倍,且表现出较少的副作用。随着世界各国对于手性药物生产和管理的规范化,对于沙美特罗替卡松粉吸入剂这种世界上最畅销的药物之一,建立昔萘酸沙美特罗对映体的手性分离分析方法,有助于进一步开展其单一对映体的相关研究,保证临床用药的安全有效。

**图1:**
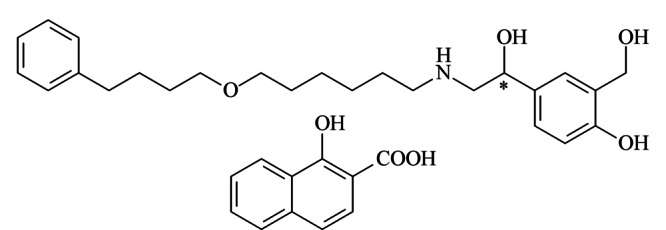
昔萘酸沙美特罗的结构式

目前,昔萘酸沙美特罗的手性分离方法主要有高效液相色谱法(high performance liquid chromatography, HPLC)^[3,4]^和超临界流体色谱法(supercritical fluid chromatography, SFC)^[5]^,但是两种方法的运行成本都比较高。毛细管电泳法(capillary electrophoresis, CE)具有操作简单、分离效率高、药品和试剂用量少、可供选择模式多等优点^[6]^,因此在手性药物分离领域应用广泛^[7,8]^。非水毛细管电泳(nonaqueous capillary electrophoresis, NACE)是CE的一个重要分支,能够分离水溶性差或在水相介质中分离效果较差的分析物,有利于提高手性选择剂的识别能力^[9,10]^。NACE中常用的手性选择剂有环糊精(cyclodextrin, CD)及其衍生物、大环抗生素、蛋白质和多糖等^[11]^。手性多羟基化合物-硼酸络合酸是一类新型离子对手性选择剂,制备简单、价格低廉,在微乳液毛细管电动色谱(microemulsion electrokinetic chromatography, MEEKC)^[12,13,14]^和NACE^[15,16,17,18]^中已成功实现了多种手性药物的良好分离。本文以L(+)-酒石酸-硼酸络合酸作为手性选择剂,采用NACE法对昔萘酸沙美特罗对映体进行分离,并开展方法学研究(包括线性与范围、检出限、定量限、精密度、准确度、稳定性等),结果表明该方法结果准确可靠,可用于沙美特罗替卡松粉吸入剂中昔萘酸沙美特罗对映体含量的测定。

## 1 实验部分

### 1.1 仪器与试剂

CL101A型高效毛细管电泳仪(北京华阳利民仪器公司),所有数据采集均在CXTH-3000色谱工作站上完成;KQ-500DE型数控超声波清洗器(昆山市超声仪器公司); BT25S型电子分析天平(北京赛多利斯科仪器公司); PHS-3C型pH计(上海理达仪器厂)。

L(+)-酒石酸(分析纯,天津市科密欧化学试剂有限公司);硼酸(分析纯,天津北方天医化学试剂厂);三乙胺和冰乙酸(分析纯,天津市光复精细化工研究所);甲醇(色谱纯,北京百灵威科技有限公司);昔萘酸沙美特罗对照品(批号100567-201502,纯度为99.9%)购自中国食品药品检定研究院;沙美特罗替卡松粉吸入剂(含昔萘酸沙美特罗50.0 μg/泡,批号为5L7P)由英国葛兰素史克公司生产。

### 1.2 溶液配制

背景缓冲溶液:取适量L(+)-酒石酸和硼酸,精密称定,置于25.0 mL量瓶中,加少量甲醇超声溶解并定容至刻度,混合均匀,0.22 μm微孔滤膜(有机系)过滤,超声脱气15.0 min。

对照品溶液:取适量昔萘酸沙美特罗对照品,精密称定,溶于甲醇中,配制成2.0 g/L的储备液,4 ℃冰箱保存,进样前用甲醇稀释到合适浓度作为对照品溶液。

样品溶液:取适量沙美特罗替卡松粉吸入剂泡囊内容物,精密称定,用甲醇超声溶解,4000 r/min下离心5.0 min,取上清液,用甲醇配制成消旋体质量浓度约为2.0 g/L的储备液,4 ℃冰箱保存,进样前用甲醇稀释到合适浓度作为样品溶液。

### 1.3 CE的条件与方法

未涂层弹性熔融石英毛细管(内径50.0 μm,总长度64.5 cm,有效长度55.5 cm,购自河北永年锐沣色谱器件有限公司),在使用前依次用甲醇冲洗10.0 min,超纯水冲洗10.0 min, 1.0 mol/L氢氧化钠溶液冲洗20.0 min,超纯水冲洗至中性,1.0 mol/L盐酸溶液冲洗20.0 min,超纯水冲洗至中性,最后用甲醇冲洗5.0 min。每次进样前,用背景缓冲溶液冲洗3.0 min。重力进样17.5 cm×10.0 s,检测波长225 nm;室温;正极进样,负极检测;运行电压20.0 kV。

## 2 结果与讨论

### 2.1 手性分离条件的优化

2.1.1 L(+)-酒石酸浓度对手性分离的影响

手性选择剂在手性分离中起着至关重要的作用。本实验在0~140.0 mmol/L范围内,考察了L(+)-酒石酸浓度对手性分离的影响。如图2所示,保持硼酸浓度为120.0 mmol/L不变,当L(+)-酒石酸的浓度由0 mmol/L逐渐增加到120.0 mmol/L时,昔萘酸沙美特罗两个对映体间的分离度(resolution, *R*_s_)总体上逐渐增大,这是由于生成手性络合酸的反应是可逆的,L(+)-酒石酸浓度的增加促进反应正向移动,有利于络合酸手性选择剂生成量的增加;当L(+)-酒石酸的浓度从120.0 mmol/L逐渐增加到140.0 mmol/L时,昔萘酸沙美特罗两个对映体间的*R*_s_开始减小。本实验最终选择120.0 mmol/L作为L(+)-酒石酸的最佳浓度。

**图2:**
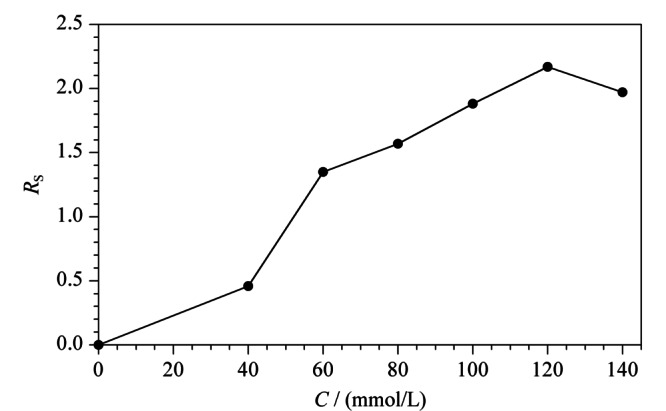
L(+)-酒石酸浓度对手性分离的影响(*n*=3)

2.1.2 硼酸浓度对手性分离的影响

保持L(+)-酒石酸浓度为120.0 mmol/L不变,本实验在0~140.0 mmol/L范围内,考察了硼酸浓度对手性分离的影响。如图3所示,当硼酸浓度由0 mmol/L逐渐增加到120.0 mmol/L时,昔萘酸沙美特罗两个对映体间的*R*_s_总体上逐渐增大,原因与L(+)-酒石酸的影响类似,硼酸浓度的增加促进了络合酸手性选择剂生成量的增加;当硼酸的浓度超过120.0 mmol/L时,昔萘酸沙美特罗两个对映体间的*R*_s_减小。本实验最终选择120.0 mmol/L作为硼酸的最佳浓度。

**图3:**
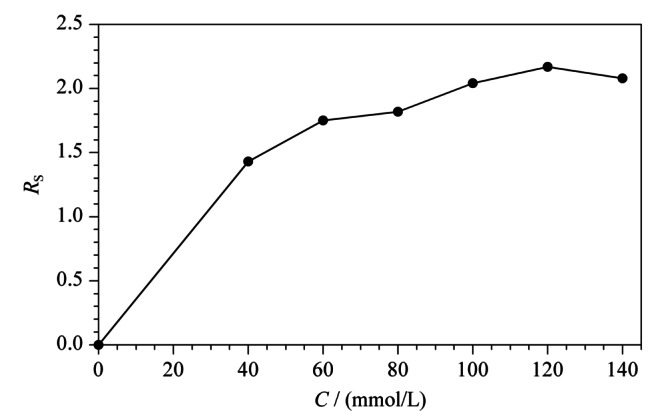
硼酸浓度对手性分离的影响(*n*=3)

2.1.3 表观pH对手性分离的影响

NACE中缓冲液的酸度用表观pH(apparent pH, pH^*^ )来表示,它是影响手性分离的重要参数之一。不同浓度的冰乙酸和三乙胺会影响缓冲溶液的pH^*^,进而影响手性药物的解离程度和手性选择剂的生成量,同时改变电渗流(electroosmotic flow, EOF)的大小,改善分离效果^[19]^。保持L(+)-酒石酸和硼酸浓度均为120.0 mmol/L不变,改变冰乙酸和三乙胺的浓度,在pH^*^ 0.53~1.02范围内考察昔萘酸沙美特罗迁移时间(migration time, *t*)和*R*_s_的变化(见图4)。随着pH^*^ 逐渐增大,EOF减小,两个对映体的*t*增加;当pH^*^ 从0.53逐渐增加到0.93时,*R*_s_达到2.18;继续增加三乙胺浓度来增大pH^*^,昔萘酸沙美特罗的两个对映体由于*t*太长,样品扩散,*R*_s_降低。本实验最终选择0.93为最佳pH^*^ 。

**图4:**
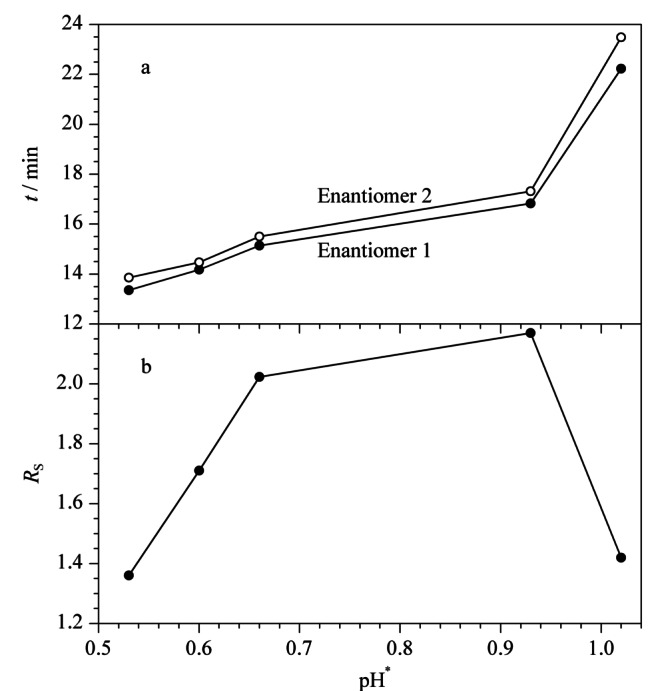
pH^*^ 对手性分离的影响(*n*=3)

本实验确定的最佳缓冲溶液为:含120.0 mmol/L L(+)-酒石酸和120.0 mmol/L硼酸的甲醇溶液(pH^*^ =0.93),其他电泳条件见1.3节。如图5所示,在优化的实验条件下,昔萘酸沙美特罗的两个对映体实现了良好分离。

**图5:**
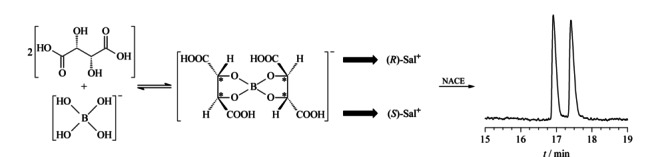
L(+)-酒石酸-硼酸络合酸作为手性选择剂的NACE法分离昔萘酸沙美特罗对映体机理示意图

### 2.2 手性分离机理

如图5所示,L(+)-酒石酸是一种手性多羟基化合物,分子结构中含有一对顺-邻二羟基,可以和硼酸在甲醇溶液中按照物质的量之比2∶1生成环状结构的L(+)-酒石酸-硼酸络合酸,这种环状结构能够限制单键的旋转,固定手性中心,使手性络合酸与两个对映体空间匹配方面的差别增大,从而提高其手性识别作用^[15,16,17,18]^。由于这个反应是可逆的,无法获得纯净的络合酸产物。在本实验的缓冲体系中,L(+)-酒石酸-硼酸络合酸解离生成络合酸阴离子,它是NACE手性分离中真正的手性选择剂;昔萘酸沙美特罗解离生成(*R*)-Sal^+^和(*S*)-Sal^+^两个阳离子,它们可以和手性络合酸阴离子分别生成暂稳态的离子对化合物,由于二者生成离子对化合物的平衡常数不同,在NACE中的表观迁移速率也不同,从而实现手性分离。

### 2.3 系统适用性试验

取对照品溶液和市售制剂样品溶液,在优化的手性分离条件下进样检测。如图6所示,昔萘酸沙美特罗两个对映体的色谱峰峰形对称,*R*_s_大于2.0,且理论塔板数、拖尾因子和重复性可满足分析要求,沙美特罗替卡松粉吸入剂中的丙酸氟替卡松不干扰分离。

**图6:**
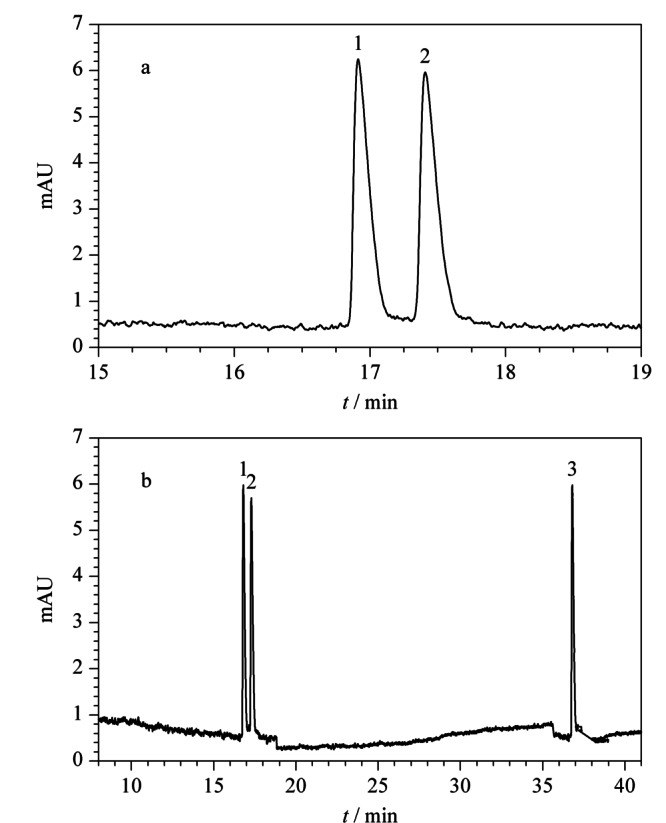
NACE系统适用性试验色谱图

### 2.4 方法学考察

2.4.1 线性与范围

精密量取2.0 g/L的昔萘酸沙美特罗对照品储备液适量,用甲醇稀释成55.0、100.0、200.0、400.0、800.0、1600.0 mg/L的系列标准溶液,在优化的电泳条件下进样检测,每份溶液平行测3次,记录每个对映体的峰面积。如表1所示,昔萘酸沙美特罗对映体1和对映体2在27.5~800.0 mg/L质量浓度范围内,与峰面积均呈现良好的线性关系,相关系数(*r*)分别为0.9991和0.9992。

**表1:** L(+)-酒石酸-硼酸络合物为手性选择剂的NACE方法的验证结果

Analyte	Regression equation	r	Linear range/ (mg/L)	LOD/ (mg/L)	LOQ/ (mg/L)	RSDs/%										
						Intra-day (n=6)				Inter-day (n=15)						
t	A		t				A
Enantiomer 1	Y=114692X+2546.1	0.9992	27.5-800.0	7.5	25.0	1.1		3.0				1.9				4.7
Enantiomer 2	Y=113497X+3106.4	0.9991	27.5-800.0	7.5	25.0	1.3		3.2				1.9				4.9

The concentration of each enantiomer was calculated as a half of its racemate. The characters of *t* and *A* represent the migration time and peak area of the enantiomer, respectively.

2.4.2 检出限与定量限

以信噪比为3(*S/N*=3)和10(*S/N*=10)确定检出限和定量限。如表1所示,昔萘酸沙美特罗对映体1和对映体2的检出限和定量限分别为7.5和25.0 mg/L。

2.4.3 精密度

取0.8 g/L的昔萘酸沙美特罗对照品甲醇溶液,在优化的电泳条件下进样检测,记录每个对映体的迁移时间和峰面积。同一日内连续进样6次,测定日内精密度;每天进样3次,连续进样5天,测定日间精密度。如表1所示,昔萘酸沙美特罗对映体1和对映体2迁移时间和峰面积的日内和日间精密度RSD值分别低于1.9%和4.9%,说明该方法的精密度良好。

2.4.4 样品溶液的稳定性

取0.8 g/L的样品溶液,分别放置0、2.0、4.0、6.0、8.0、10.0、12.0和24.0 h之后,在优化的电泳条件下进样检测,每份溶液平行测3次,记录每个对映体的迁移时间和峰面积。结果表明,样品溶液中昔萘酸沙美特罗对映体1和对映体2迁移时间的RSD分别为1.3%和1.1%,峰面积的RSD分别为3.9%和4.5%,说明该样品溶液在24.0 h内稳定性良好。

2.4.5 加标回收率

精密量取183.5 mg/L的样品溶液0.2 mL共9份,平均分为3组,使用微量进样器分别加入15.0、17.5和20.0 μL昔萘酸沙美特罗对照品储备液(2.0 g/L),获得低、中和高3组加标样品溶液,每组3份样品,在优化的电泳条件下进样检测,每份溶液平行测3次,记录每个对映体的峰面积。如表2所示,昔萘酸沙美特罗对映体1和对映体2的加标回收率分别为99.3%~100.1%和99.8%~100.4%, RSD在1.2%~1.9%之间,说明该方法的准确度良好。

**表2:** 沙美特罗替卡松粉吸入剂中昔萘酸沙美特罗对映体的加标回收率(*n*=3)

Analyte	Background/ μg	Added/ μg	Found/ μg	Recovery/ %	RSD/ %
Enantiomer 1	36.7	30.0	66.8	100.1	1.8
	36.7	35.0	71.5	99.4	1.2
	36.7	40.0	76.4	99.3	1.8
Enantiomer 2	36.7	30.0	66.9	100.4	1.9
	36.7	35.0	71.6	99.8	1.4
	36.7	40.0	76.8	100.2	1.7

### 2.5 实际样品测定

取适量市售沙美特罗替卡松粉吸入剂6份,配制样品溶液,对其昔萘酸沙美特罗对映体的含量进行测定。在优化的电泳条件下进样检测,每份溶液平行测定3次,记录每个对映体的峰面积,计算含量。根据公式“标示量百分含量=(测得量/标示量)×100%”计算每个对映体的标示量百分含量。本实验所使用的沙美特罗替卡松吸入粉剂中昔萘酸沙美特罗的标示量为50.0 μg/泡,两个对映体的标示量分别按照25.0 μg/泡计算。结果显示,对映体1和对映体2的标示量百分含量均为98.7%, RSD分别为2.5%和2.7%,测定结果的重复性良好。

## 3 结论

L(+)-酒石酸-硼酸络合酸价廉易得,本文以其作为手性选择剂建立了NACE手性分离方法,成功实现了昔萘酸沙美特罗对映体的基线分离。该方法操作简单、快速、结果准确可靠,可用于市售沙美特罗替卡松粉吸入剂中昔萘酸沙美特罗对映体含量的测定。

## References

[1] Paik J, Scott L J, Pleasants R A. Clin Drug Invest, 2018,38:463 10.1007/s40261-018-0644-2 29582249

[2] Jacobson G A, Hostrup M, Narkowicz C K, et al. Drug Test Anal, 2017,9(8):1262 28033454 10.1002/dta.2131

[3] Kim K H, Yun H W, Kim H J, et al. Arch Pharmacal Res, 1998,21(2):212 10.1007/BF02974030 9875433

[4] Yang Y J, Hou M Q. Chinese Journal of Pharmaceutical Analysis, 2001,21(2):97

[5] Mikushina I V, Bazarnova N G, Parenago O O, et al. J Sib Fed Univ Chem, 2019,3(12):310

[6] Liu M X, Li X J, Bai Y, et al. Chinese Journal of Chromatography, 2020,38(3):317 34213211 10.3724/SP.J.1123.2019.10019

[7] Du Y X, Yan Z. Northwest Pharmaceutical Journal, 2015,30(6):766

[8] Li X, Wang W, Tan X X, et al. Chinese Journal of Analysis Laboratory, 2017,36(2):138

[9] Wang C Y, Sun W Y, Lü H W, et al. Physical Testing and Chemical Analysis (Part B: Chemical Analysis), 2019,55(10):1235

[10] Ou W L, Li Y J, Shi D D, et al. Chinese Journal of Chromatography, 2015,33(2):152 25989687 10.3724/sp.j.1123.2014.11006

[11] Ali I, Sanagi M M, Aboul-Enein H Y. Electrophoresis, 2014,35(7):926 23913555 10.1002/elps.201300222

[12] Hu S Q, Chen Y L, Zhu H D, et al. J Chromatogr A, 2009,126(45):7932 10.1016/j.chroma.2009.09.036 19782374

[13] Hu S Q, Chen Y L, Zhu H D, et al. J Chromatogr A, 2010,1217(34):5529 20638068 10.1016/j.chroma.2010.06.063

[14] Hu S Q, Lu W J, Ma Y H, et al. Electrophoresis, 2013,34(2):260 23161242 10.1002/elps.201200150

[15] Wang L J. Chiral Separation Methods and Protocols. New York: Humana Press, 2019

[16] An N, Wang L J, Zhao J J, et al. Anal Methods, 2016,8:1127

[17] Wang L J, Liu X F, Lu Q N, et al. J Chromatogr A, 2013,1284:188 23434083 10.1016/j.chroma.2013.02.006

[18] Lv L L, Wang L J, Li J, et al. J Pharm Biomed Anal, 2017,145:399 28719814 10.1016/j.jpba.2017.06.044

[19] Gong Z S, Duan L P, Tang A N. Microchim Acta, 2015,182(7/8):1297

